# Control Group Outcomes in Trials of Psilocybin, SSRIs, or Esketamine for Depression: A Meta-Analysis

**DOI:** 10.1001/jamanetworkopen.2025.24119

**Published:** 2025-07-30

**Authors:** Fredrik Hieronymus, Evana López, Helena Werin Sjögren, Johan Lundberg

**Affiliations:** 1 Institute of Neuroscience and Physiology, University of Gothenburg, Gothenburg, Sweden; 2 Department of Affective Disorders, Aarhus University, Aarhus, Denmark; 3 Centre for Psychiatry Research, Karolinska Institute & Stockholm Health Care Services, Region Stockholm, Sweden; 4 Sahlgrenska University Hospital, Gothenburg, Sweden

## Abstract

**Importance:**

Psilocybin has demonstrated rapid and sustained antidepressant efficacy, with acute-phase effect sizes often more than double those for conventional antidepressants. However, concerns have been raised that high rates of functional unblinding in combination with trial participants with positive expectations of psychedelic use might bias treatment outcomes.

**Objective:**

To compare outcomes for patients receiving control treatments in randomized clinical trials of psilocybin for depression with control treatment outcomes from trials of selective serotonin reuptake inhibitors (SSRIs) and esketamine.

**Data Sources:**

Two previous meta-analyses and 1 US Food and Drug Administration review published between March 2019 and December 2024 were used to identify double-blind trials on adult major depressive disorder (MDD) or treatment-resistant depression (TRD) that had a relevant control treatment arm and used the Montgomery-Åsberg Depression Rating Scale (MADRS) for symptom rating.

**Study Selection:**

Following the Preferred Reporting Items for Systematic Reviews and Meta-Analyses reporting guideline, trials of psilocybin for MDD and TRD, esketamine for TRD, and a selective serotonin reuptake inhibitor (SSRI) for MDD were selected. Studies that included only individuals aged younger than 18 years or older than 65 years, used a crossover design, or had a duration less than 2 weeks were excluded.

**Data Extraction and Synthesis:**

All authors assessed the 3 reviews for includable trials. Three authors independently extracted data for all trials, with disagreements resolved by consensus discussion. Data were pooled using random-effects models.

**Main Outcomes and Measures:**

Standardized mean change (SMC) in MADRS scores from baseline to up to 6 weeks after randomization was used to assess within-group effect sizes, and standardized mean difference (SMD) was used to assess between-group effect sizes. Omnibus Test of Moderators (QM) was used to test whether the study population significantly moderated effect sizes.

**Results:**

The study included 17 trials: 4 of psilocybin (n = 373), 2 of esketamine (n = 573), and 11 of SSRIs (n = 4014). Pretreatment to posttreatment SMCs (SEMs) were 1.21 (0.15) for psilocybin, 1.28 (0.06) for SSRIs, and 1.43 (0.15) for esketamine and were 0.50 (0.15), 1.00 (0.08), and 1.12 (0.17) for their respective control treatments. Study population was a significant moderator of between-group SMDs (QM, 10.7; *df*, 2; *P* = .005) and pre– to post–control treatment SMCs (QM, 10.4; *df*, 2; *P* = .005) but not of pre– to post–active treatment SMCs (QM, 1.21; *df*, 2; *P* = .55). MADRS response rates for control treatments in SSRI trials were 14 percentage points higher than in psilocybin trials and in esketamine trials were 23 percentage points higher than in psilocybin trials. Dropout rates for psilocybin (active treatment: 10 of 186 [5%]; control: 20 of 187 [11%]) and esketamine (active treatment: 43 of 349 [12%]; control: 18 of 224 [8%]) were similar and considerably lower than for SSRIs (active treatment: 866 of 2694 [32%]; control: 467 of 1320 [35%]).

**Conclusions and Relevance:**

In this meta-analysis of control treatment outcomes in trials of psilocybin, SSRIs, or esketamine for depression, participants receiving control treatment in psilocybin trials had significantly less improvement in depression ratings than participants receiving control treatment in trials of SSRIs or esketamine. This might indicate that psilocybin’s antidepressant efficacy is overestimated compared with that of SSRIs and esketamine.

## Key Points

**Question:**

Are there differences in depression outcomes among patients who receive control treatments in trials of psilocybin, selective serotonin reuptake inhibitors (SSRIs), or esketamine for depression?

**Findings:**

In this meta-analysis of 17 acute-phase randomized clinical trials of psilocybin (n = 373), esketamine (n = 573), or SSRIs (n = 4014), patients receiving control treatments in psilocybin trials had significantly worse depression outcomes compared with those receiving control treatments in trials of esketamine or SSRIs. There were larger between-treatment differences for psilocybin than for esketamine or SSRIs.

**Meaning:**

The poor control treatment outcomes in psilocybin trials suggest that it may not be as broadly effective for depression as estimated.

## Teaser

This meta-analysis compares depression rating outcomes among patients receiving control treatments in randomized clinical trials of psilocybin vs trials of selective serotonin reuptake inhibitors (SSRIs) and esketamine for depression.

## Introduction

Antidepressant efficacy is typically established by comparing a candidate antidepressant with some control treatment for 4 to 12 weeks. The response to control treatments in depression varies but is often substantial.^1^ Jones and coworkers^2^ reported the average pretreatment to posttreatment effect size across a range of control treatments for treatment-resistant depression (TRD) to be 1.05, and Cuijpers and colleagues^3^ found pretreatment to posttreatment effect sizes of 0.64 for care as usual and 0.37 for waiting list controls in a meta-analysis of psychotherapies for depression.

Psilocybin is a novel candidate antidepressant with a unique mechanism of action that has demonstrated substantial acute-phase efficacy in depression, with effect sizes often more than double those for conventional antidepressants.^4^ However, control treatment response rates have been noted to be lower than expected in some trials.^5^ Psilocybin in trials for depression has been given in psychedelic doses, which risks making functional unblinding both immediate and prevalent.^6^ Furthermore, since administration is usually supervised, unblinding may also extend to study personnel.^7^ If control treatment response rates are low across the board for psilocybin trials, it could indicate that efficacy is overestimated (eg, via functional unblinding and nocebo effects).

To assess whether low control treatment efficacy is a general phenomenon in trials of psilocybin for depression, we performed an indirect comparison of outcomes on the Montgomery-Åsberg Depression Rating Scale (MADRS) for 3 trial populations: (1) psilocybin trials on major depressive disorder (MDD) and TRD, (2) esketamine trials on TRD, and (3) selective serotonin reuptake inhibitor (SSRI) trials on MDD. Since the hypothesis was that control treatment outcomes may differ systematically depending on which active drug is trialed, there was no common treatment through which indirect evidence might be contrasted (ie, inert placebo might work differently in an SSRI trial than it does in a psilocybin trial). For this reason, the analyses were undertaken using conventional meta-analysis techniques rather than using network meta-analysis.^8^

## Methods

### Data Acquisition

In this meta-analysis, double-blind trials on adult MDD or TRD with a relevant control treatment arm (inert placebo, low-dose psilocybin, or niacin) and that had used the MADRS for symptom rating were included from 3 reviews published between March 2019 and December 2024.^9,10,11^ We followed the Preferred Reporting Items for Systematic Reviews and Meta-analyses (PRISMA) reporting guideline. All authors assessed the 3 reviews for includable trials. F.H., E.L., and H.W.S. independently extracted data for all trials, with disagreements resolved by consensus discussion. Studies exclusively including individuals aged younger than 18 years or older than 65 years, studies using crossover designs, and studies with a duration of less than 2 weeks were excluded. Psilocybin trials were collected from a meta-analysis by Freitas and colleagues,^9^ esketamine trials were taken from the corresponding US Food and Drug Administration review,^10^ and SSRI trials were included from a meta-analysis of individual participant data by Hieronymus and colleagues.^11^ Data were pooled using random-effects models.

### Statistical Analysis

Within-group pretreatment to posttreatment effect sizes for control treatment and active treatment arms, respectively, and between-group (active treatment vs control treatment) effect sizes were calculated for all trials. Within-group effect sizes were calculated as standardized mean change (SMC) in MADRS scores using raw score standardization.^12^ Since SDs are usually larger at the end point than at baseline^13^ and since between-group effect sizes are normalized to the pooled end point SD, SMCs were normalized to the end point SD of the corresponding group. For 1 psilocybin study,^14^ baseline MADRS scores were not available. The pretest score for that study was imputed as the mean of all other psilocybin arms.

The correlation between preintervention and postintervention measures was set to 0 for all trials. When using raw score standardization, the pretreatment to posttreatment correlation is not used for calculating the effect size, but it affects the SE of the effect size. Specifically, SEs get larger the closer the correlation coefficient gets to −1 and smaller the closer the correlation coefficient gets to 1.^12^ Since preintervention and postintervention scores in depression are usually positively correlated (patients who begin with high scores tend to also end with high scores),^13^ assuming no correlation should yield conservative estimates. This SMC specification is functionally identical to calculating the standardized mean difference (SMD) between the preintervention and postintervention measurements using either only the control treatment arm SD or only the active treatment arm SD for normalization. SMDs normalized to the pooled SD were used for between-group effect sizes.

The 3 effect sizes (active treatment SMC, control treatment SMC, and active treatment vs control treatment SMD) were then meta-regressed with study population (study of psilocybin, esketamine, or SSRI) as the sole variable. The outcomes of interest were the pooled effect sizes for each study group and the result of the Omnibus Test of Moderators (QM) (ie, whether the study population significantly moderated effect sizes) for the 3 outcomes and the percentage of variance explained (*R*^2^) by models including the study population as a variable. We also calculated pooled MADRS response rates (≥50% reduction compared with baseline) and dropout rates. These are presented descriptively.

R, version 4.3.3 (R Project for Statistical Computing) was used for all analyses. Meta-analyses were performed using the metafor package, version 4.6.0, in R, and effect sizes (SMC using raw score standardization and SMD) were calculated using the escalc function in the metafor package; analyses were run using the rma function in the metafor package. Two-sided *P* <.05 was considered significant.

## Results

**Figure 1.**
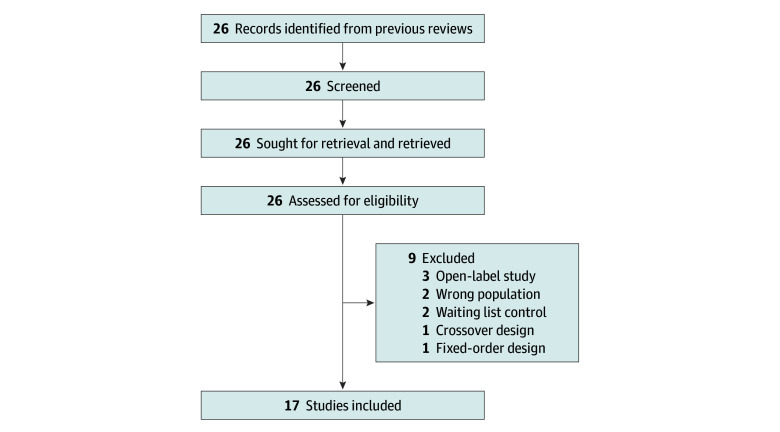
Study Identification and Screening

Four trials of psilocybin (n = 373),^5,14,15,16^ 2 trials of esketamine (n = 573),^17,18^ and 11 trials of SSRIs (n = 4014)^19,20,21,22,23,24,25,26,27,28,29^ were included. Study identification and screening are detailed in Figure 1, and baseline and end point scores for all included trials are provided in Table 1.

**Table 1.** Baseline and End Point MADRS Scores for Individual Studies

Study	Time from baseline to evaluation, wk	Participants, No.		MADRS score, mean (SD)				Years conducted
		Active treatment	Placebo	Baseline		End point		
				Active treatment	Placebo	Active treatment	Placebo	
**Psilocybin**								
Carhart-Harris et al,^14^ 2021	6	30	29	30.6 (5.7)^a^	30.6 (5.7)^a^	16.2 (9.3)	23.4 (9.2)	2019-2020
Goodwin et al,^16^ 2022	6	79	79	31.9 (5.4)	32.7 (6.2)	20.4 (13.3)	26.4 (13.3)	2019-2021
Raison et al,^5^ 2023	6	51	53	35.5 (5.7)	35.0 (4.5)	16.4 (13.1)	28.2 (13.7)	2019-2022
von Rotz et al,^15^ 2022	2	26	26	24.3 (5.1)	24.1 (7.1)	11.2 (9.2)	20.7 (10.1)	2019-2021
**Esketamine**								
S3001^17^	4	233	113	37.6 (5.2)	37.5 (6.2)	18.7 (14.0)	22.7 (15.1)	2015-2018
S3002^18^	4	116	111	37.0 (5.7)	37.3 (5.7)	16.2 (13.3)	20.5 (13.1)	2015-2017
**SSRI**								
GSK/001^19^	6	25	25	29.9 (4.5)	30.1 (4.2)	17.1 (7.0)	19.2 (7.4)	1984-1985
GSK/002^20^	6	170	171	27.9 (5.2)	28.2 (5.3)	16.5 (10.0)	21.4 (10.0)	1985-1987
GSK/003^21^	6	241	244	31.1 (5.3)	31.4 (5.4)	20.1 (10.4)	25.7 (10.2)	1985-1986
GSK/009^22^	6	421	53	28.7 (6.1)	27.5 (6.5)	17.3 (10.2)	17.8 (10.4)	1985-1986
GSK/785^23^	6	403	105	31.1 (4.8)	31.2 (4.8)	17.9 (10.8)	19.9 (10.5)	2001
LB/89303^25^	6	134	66	33.0 (7.4)	33.3 (8.3)	15.9 (13.7)	17.6 (12.9)	1989-1990
LB/89306^26^	6	185	88	32.0 (7.0)	33.1 (6.8)	15.0 (12.5)	17.6 (14.1)	1989-1991
LB/91206^27^	6	521	129	27.5 (5.6)	27.1 (6.0)	15.7 (10.4)	17.7 (10.2)	1992-1993
LB/99001^28^	6	188	189	29.2 (4.2)	28.7 (3.7)	14.6 (8.9)	18.0 (9.2)	1999-2000
LB/99003^29^	6	314	154	29.1 (4.2)	28.7 (4.0)	15.9 (8.5)	17.7 (9.2)	1999-2000
PZ/881^24^	6	92	96	27.9 (3.5)	27.2 (3.9)	14.7 (10.0)	16.1 (9.2)	1989-1990

Abbreviations: GSK, GlaxoSmithKline; LB, H. Lundbeck; MADRS, Montgomery-Åsberg Depression Rating Scale; PZ, Pfizer; SSRI, selective serotonin reuptake inhibitor.

^a^ Mean and SD imputed as the arithmetic mean from the other psilocybin and psilocybin control treatment arms.

**Figure 2.**
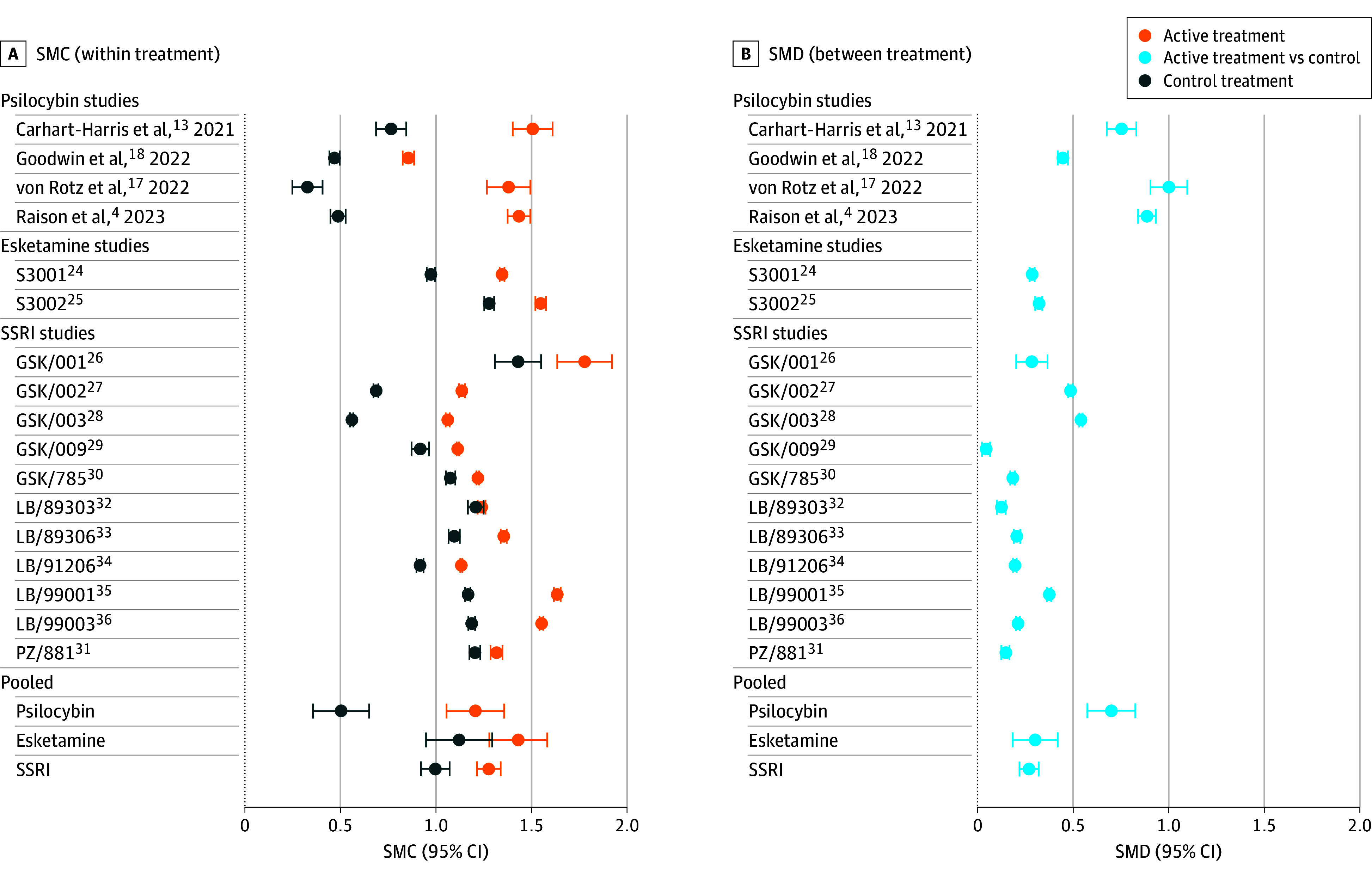
Within- and Between-Group Effect Sizes for Psilocybin, Selective Serotonin Reuptake Inhibitors (SSRIs), and Esketamine Error bars indicate plus or minus 1 SE. SMC indicates standardized mean change; SMD, standardized mean difference.

Pretreatment to posttreatment effect sizes (SMC [SEM]) were 1.21 (0.15) for psilocybin, 1.43 (0.15) for esketamine, and 1.28 (0.06) for SSRIs and were 0.50 (0.15), 1.12 (0.17), and 1.00 (0.08), respectively, for their corresponding control treatments (Figure 2). Pretreatment to posttreatment SMC differences were thus 0.71 for psilocybin, 0.29 for esketamine, and 0.28 for SSRIs, which corresponded with the actual between-group effect sizes (SMD [SEM]): 0.70 (0.12), 0.30 (0.12), and 0.27 (0.05), respectively. Study population (psilocybin, SSRIs, or esketamine) was a significant moderator of between-group effect sizes (QM, 10.7; *df*, 2; *P* = .005) and of pre– to post–control treatment effect sizes (QM, 10.4; *df*, 2; *P* = .005) but not of active treatment effect sizes (QM, 1.21; *df*, 2; *P* = .55). Models including study population as a moderator explained 40.9% of the variance (*R*^2^) in pre– to post–control treatment outcomes, 34.8% of the variance in between-group outcomes, and 0% of the variance in pre– to post–active treatment outcomes.

Pooled response rates for active treatment arms were 89 of 186 (48%) for psilocybin, 181 of 349 (52%) for esketamine, and 1245 of 2694 (46%) for SSRIs. The corresponding control treatment response rates were 35 of 187 (19%) for psilocybin, 94 of 224 (42%) for esketamine, and 433 of 1320 (33%) for SSRIs. Control treatment response rates were thus 23 percentage points higher for esketamine than for psilocybin and 14 percentage points higher for SSRIs than psilocybin. Dropout rates in psilocybin trials (active arms: 10 of 186 [5%]; control treatment arms: 20 of 187 [11%]) were similar to those in esketamine trials (active arms: 43 of 349 [12%]; control treatment arms: 18 of 224 [8%]), whereas SSRI trials had markedly higher dropout rates (active arms: 866 of 2694 [32%]; control treatment arms: 467 of 1320 [35%]). Response and dropout rates for individual trials are provided in Table 2.

**Table 2.** Response and Dropout Rates for Individual Studies

Study	Dropout rate, No./total No. (%)		Response rate, No./total No. (%)	
	Active treatment	Placebo	Active treatment	Placebo
**Psilocybin**				
Carhart-Harris et al,^14^ 2021	2/30 (7)	0/29	19/30 (63)^a^	6/29 (21)
Goodwin et al,^16^ 2022	5/79 (6)	10/79 (13)	26/79 (33)	16/79 (20)
Raison et al,^5^ 2023	1/51 (2)	9/53 (17)	29/51 (57)^a^	9/53 (17)^a^
von Rotz et al,^15^ 2022	2/26 (8)	1/26 (4)	15/26 (58)	4/26 (15)
Pooled	10/186 (5)	20/187 (11)	89/186 (48)	35/187 (19)
**Esketamine**				
S3001^17^	25/233 (11)	6/113 (5)	112/233 (48)^a^	42/113 (37)^a^
S3002^18^	18/116 (16)	12/111 (11)	69/116 (59)^a^	52/111 (47)^a^
Pooled	43/349 (12)	18/224 (8)	181/349 (52)^a^	94/224 (42)^a^
**SSRIs**				
GSK/001^19^	13/25 (52)	11/25 (44)	8/25 (32)	11/25 (44)
GSK/002^20^	84/170 (49)	95/171 (56)	69/170 (41)	38/171 (22)
GSK/003^21^	121/241 (50)	151/244 (62)	87/241 (36)	42/244 (17)
GSK/009^22^	180/421 (43)	23/53 (43)	167/421 (40)	17/53 (32)
GSK/785^23^	150/403 (37)	31/105 (30)	179/403 (44)	38/105 (36)
LB/89303^25^	34/134 (25)	20/66 (30)	79/134 (59)	34/66 (52)
LB/89306^26^	53/185 (29)	25/88 (28)	110/185 (59)	50/88 (57)
LB/91206^27^	168/521 (32)	43/129 (33)	256/521 (49)	43/129 (33)
LB/99001^28^	21/188 (11)	24/189 (13)	99/188 (53)	69/189 (37)
LB/99003^29^	13/314 (4)	9/154 (6)	143/314 (46)	55/154 (36)
PZ/881^24^	29/92 (32)	35/96 (36)	48/92 (52)	36/96 (38)
Pooled	866/2694 (32)	467/1320 (35)	1245/2694 (46)	433/1320 (33)

Abbreviations: GSK, GlaxoSmithKline; LB, H. Lundbeck; PZ, Pfizer; SSRI, selective serotonin reuptake inhibitor.

^a^ Corrected for intention to treat.

### Post Hoc Analysis

We originally opted to exclude 3 esketamine studies conducted in patients with depression and acute suicidality (ASPIRE 1,^30^ ASPIRE 2,^30^ and SUI2001^31^) since there were no psilocybin or SSRI studies in that population. When we performed a separate meta-analysis of those studies as a post hoc analysis, the mean MADRS decrease from baseline for esketamine control treatment in participants with depression and acute suicidality was 22.9 points, which corresponded to an SMC (SEM) of 1.87 (0.12).

## Discussion

This meta-analysis found that control treatment depression scores in psilocybin trials were lower than those usually seen in antidepressant trials^2,3^ and significantly lower than those for esketamine and SSRIs. In absolute terms, effect sizes in control treatment arms (0.50) in psilocybin trials were closer to those in waiting list control (0.37) and care as usual (0.64) arms in psychotherapy trials^3^ than to those in pill placebo arms in antidepressant trials (1.05).^1,2^ Conversely, pretreatment to posttreatment effect sizes in active treatment arms did not differ significantly between study populations (Figure 2). MADRS response rates for patients receiving control treatment were 14 percentage points lower in psilocybin trials than in SSRI trials and 23 percentage points lower in psilocybin trials than in esketamine trials (Table 2).

The poor performance of control treatments in psilocybin trials is likely caused by 1 of 2 factors: psilocybin trials have recruited patients who are unlikely to respond to control treatment or the psilocybin trialing process applied is less likely to induce control treatment response. In other words, assuming that the observed differences are not explained by chance, psilocybin trials must systematically differ from trials of SSRIs and esketamine either in the patient makeup or in some relevant methodologic aspect.

One potential systematic methodologic difference is that blinding is more difficult to maintain with psilocybin administration compared with SSRI or esketamine administration^6,7^ and that control treatment in psilocybin trials consequently functions more akin to a waiting list control than to a conventional placebo control in terms of expectancy effects. While this hypothesis aligns with a much discussed difference between the studied treatments,^6,7^ the analyses herein reported were indirect and, thus, can provide little guidance as to why the 3 trial populations differed. Any number of potential systematic differences (eg, regarding funding, publication year, number of study sites, inclusion and exclusion criteria, or concomitant treatments^32,33,34^) among the 3 studied trial populations may exist, and any one of those could be what underlies the differences in control treatment outcomes. Alternatively formulated, the present results showed that the sample average treatment effects (ATEs) differed between psilocybin control treatment and esketamine and SSRI control treatments. However, the data did not allow us to determine the specific factors that explained the observed sample ATE differences or the extent to which the sample ATEs generalized to the population ATE for the respective treatments.

Adding to the uncertainty, while unblinding has been shown to be prevalent in some psilocybin studies,^7^ rates of unblinding were not assessed in the included psilocybin^5,14,15,16^ and esketamine^10^ trials and likely were not assessed in any of the included SSRI studies. The extent to which unblinding occurred in the different study groups was thus not possible to discern from the available data. Esketamine, similar to psilocybin, is administered in the presence of health care professionals and has immediate adverse effects, which may lead to unblinding.^10^ That esketamine was associated with a degree of control treatment improvement similar to that of SSRIs thus suggests that psilocybin has a higher risk for unblinding than esketamine or that esketamine and psilocybin trials differ in some other methodologically relevant aspect.

When designing this study, we reasoned that the available trial data would not be sufficient to determine with certainty which factor or factors were causative of any putative differences in control treatment outcomes between study populations. We assumed that owing to the limited number of relevant studies available for psilocybin and esketamine, at least some potentially relevant confounders would cluster to make it impossible to separate them from one another. For example, most SSRI trials were undertaken during the 1980s and 1990s, whereas all psilocybin trials were published after 2020 (Table 1). Consequently, separating the effects of time from the effects of partaking in an SSRI trial is not possible, and the same likely holds true for many other confounders of potential importance.

While not much can thus be said about which specific factors cause the differences in control treatment outcomes, 1 factor that can be ruled out with some confidence is differential dropout.^35^ Esketamine trials were typically shorter than psilocybin trials and had comparable dropout rates but showed a larger control treatment response, whereas SSRI trials were of similar duration to psilocybin trials but had higher noncompletion rates; however, they also showed a larger control treatment response. Relatedly, the high SSRI dropout rates seen for both control treatment and active treatment are likely explained in part by SSRI trials being older (Table 1) and older trials in general having higher dropout rates.^34^ Another factor that may have contributed to differences in control treatment outcomes is that psilocybin and esketamine are both administered on site, whereas SSRIs are not.

While realizing that we would not be able to account for many important confounders, we aspired to not unnecessarily introduce additional ones. Thus, we excluded studies with short duration (<2 weeks) since there are almost no such SSRI studies; we similarly did not include studies involving only children or individuals aged older than 65 years since to our knowledge, there are no studies of psilocybin for depression in these populations. In the same vein, we did not include the 2 ASPIRE studies^30^ or SUI2001^31^ of esketamine, which were conducted in patients with depression and acute suicidality, since patients with acute suicidality are typically excluded from both SSRI and psilocybin trials.

In a meta-analysis of those trials,^30,31^ the mean MADRS decrease from baseline for control treatment was 22.9 points compared with 14.9 and 16.8 points for S3001^17^ and S3002,^18^ respectively,^10^ corresponding to an SMC (SEM) of 1.87 (0.12). One possible explanation for why control treatment was associated with a large degree of improvement in these studies^30,31^ could be that patients who present with acute suicidality may be more likely to be anxious, agitated, and potentially more prone to endorse greater symptom severity. Thus, when the acute distress abates, symptom severity likely lessens considerably irrespective of which specific treatment is provided.

While there is considerable uncertainty regarding the factors contributing to the divergent control treatment outcomes, of note, of the 3 studied compounds, psilocybin is the divergent one that meshes poorly with results from similar trials of other compounds. Thus, the outcomes herein observed for SSRI control treatments (SMC, 1.00) and esketamine control treatments (SMC, 1.12) agree with results from previous assessments of pill placebo control treatments.^1,2^ They also correspond to the mean (SEM) SMC (1.11 [0.02]) for the 242 placebo control groups with useable data included in the 2018 network meta-analysis by Cipriani and colleagues (our analysis).^36^ The mean SMC of 0.50 for psilocybin control treatments in this study, however, was below that commonly observed for pill placebo groups in depression and is more in line with outcomes from waiting list– and care as usual–controlled trials of psychotherapy.^3^

Irrespective of the causes, the small improvement rate for psilocybin control treatments in this study’s data (collected up to 6 weeks after randomization) suggests that psilocybin may not be as broadly effective for depression as estimated. If psilocybin trials have recruited patients who are less likely to respond to control treatment, it remains to be shown that psilocybin is highly effective also in the presumably larger population that tends to respond to control treatment. If a methodologic factor common to psilocybin trials (eg, that control treatment acts more like a waiting list control than a pill placebo control^7^) results in poorer control treatment outcomes, the acute antidepressant effects of psilocybin may be overestimated also for the comparatively narrow population of patients with depression for whom it has been trialed.

### Limitations

The primary limitation of the present study is that it can only conclude that control treatment outcomes differed between trial populations but could not inform the reasons for the difference. In addition, the available psilocybin literature is small and heterogenous, and the reference groups (esketamine and SSRIs) are not exhaustive. Since the control group results for esketamine and SSRIs were in line with those from other studies,^1,2^ the latter limitation is unlikely to have had a major impact. Similarly, the study by Carhart-Harris and colleagues^14^ compared 25 mg of psilocybin with combined treatment with 1 mg of psilocybin and the SSRI escitalopram. We included this study (1) since an SSRI should be at least as effective as an inert placebo and (2) since the study had the largest pre– to post–control treatment score change of the 4 psilocybin trials^5,14,15,16^ (Table 1). Excluding this study would hence yield a lower mean control treatment outcome for psilocybin. Sensitivity analyses omitting this trial did not alter the results.

## Conclusions

This meta-analysis found that participants receiving control treatment in psilocybin trials had significantly less improvement in depression ratings than participants receiving control treatment in trials of SSRIs or esketamine. The low response to control treatment suggests that psilocybin may not be as broadly effective for depression as estimated. Future studies should strive toward better understanding of which factors moderate control treatment outcomes in psilocybin trials, such as by trialing multiple control treatments and/or by recruiting study participants with positive expectations of the control treatment also.

## Notes

Supplement.
Data Sharing Statement
