# Error in Conflict of Interest Disclosures

**DOI:** 10.1001/jamanetworkopen.2025.36707

**Published:** 2025-09-11

In the Original Investigation titled “Control Group Outcomes in Trials of Psilocybin, SSRIs, or Esketamine for Depression: A Meta-Analysis,” published on July 30, 2025, in the Conflict of Interest Disclosures for Dr Lundberg, Osmond Labs was changed to the foundation Norrsken Mind. This article has been corrected online.^1^
