# Supplementary material for: Control Group Outcomes in Trials of Psilocybin, SSRIs, or Esketamine for Depression: A Meta-Analysis
Source: JAMA Netw Open. 2025 Jul 30;8(7):e2524119. doi: 10.1001/jamanetworkopen.2025.24119 (PMC12311713; doi:10.1001/jamanetworkopen.2025.24119)
Supplement: Supplement. — Data Sharing Statement [file jamanetwopen-e2524119-s001.pdf]

Data Sharing Statement

Hieronymus. Control Group Outcomes in Trials of Psilocybin, SSRIs, or Esketamine for Depression. *JAMA Netw Open*. Published July 30, 2025.
doi:10.1001/jamanetworkopen.2025.24119

Data
Data available: No

Additional Information
Explanation for why data not available: The data used for this study is publicly available in the submitted manuscript and/or from other publicly available sources.
